# Transcriptomic and metabolomic analysis of recalcitrant phosphorus solubilization mechanisms in *Trametes gibbosa*

**DOI:** 10.3389/fmicb.2025.1520459

**Published:** 2025-02-04

**Authors:** Yulan Chen, Akasha Farooq, XieLuyao Wei, Leitao Qin, Yong Wang, Lingzi Zhang, Quanju Xiang, Ke Zhao, Xiumei Yu, Qiang Chen, Xue Gao, Tashi Nyima, Petri Penttinen, Yunfu Gu

**Affiliations:** ^1^Department of Microbiology, College of Sources, Sichuan Agricultural University, Chengdu, China; ^2^Institute of Agricultural Resources and Environmental Science, Tibet Academy of Agricultural and Animal Husbandry Sciences, Lhasa, Tibet, China

**Keywords:** phosphate solubilizing fungi, transcriptomic analysis, metabolomic analaysis, phosphorus solubilization mechanism, bio-phosphate fertilizer

## Abstract

**Introduction:**

Phosphorus (P) is a crucial growth-limiting nutrient in soil, much of which remains challenging for plants to absorb and use. Unlike chemical phosphate fertilizers, phosphate-solubilizing microorganisms (PSMs) offer a means to address available phosphorus deficiency without causing environmental harm. PSMs possess multiple mechanisms for phosphorus solubilization. Although the phosphorus-solubilizing mechanisms of phosphate-solubilizing bacteria (PSB) have been well characterized, the mechanisms utilized by phosphate-solubilizing fungi (PSF) remain largely unexplored.

**Methods:**

This study isolated a PSF strain, *Trametes gibbosa* T-41, from soil and evaluated its phosphorus solubilizing capacity with organic (calcium phytin; Phytin-P) and inorganic (tricalcium phosphate; Ca-P) phosphorus sources. The phosphorus solubilization, enzyme activity, and organic acid production of T-41 were measured. And the P-solubilizing mechanism conducted by transcriptomic and metabolomic analyses.

**Results and discussion:**

T-41 exhibited varying phosphorus solubilizing capacity when grown with organic (calcium phytin; Phytin-P) and inorganic (tricalcium phosphate; Ca-P) phosphorus sources (109.80 ± 8.9 mg/L vs. 57.5 ± 7.9 mg/L, *p* < 0.05). Compared with the Ca-P treatment, T-41 demonstrated a stronger alkaline phosphatase (ALP) production capacity under Phytin-P treatment (34.5 ± 1.2 μmol/L/h vs. 19.8 ± 0.8 μmol/L/h, *p* < 0.05). Meanwhile, the production of oxalic acid, maleic acid, and succinic acid was higher under Phytin-P treatment (*p* < 0.05). Transcriptomic and metabolomic analysis revealed that different phosphorus sources altered metabolic pathways such as galactose metabolism, glyoxylate and dicarboxylic acid metabolism, and ascorbate and aldolate metabolism. Key metabolites like myo-inositol, 2-oxoglutarate, and pyruvate were found to impact the performance of *T. gibbosa* T-41 differently under the two P sources. Notably, synthesis in Ca-P vs. Pytin-P, T-41 upregulated genes involved in myo-inositol synthesis, potentially enhancing its P-solubilizing ability. These results provide new insights into the molecular mechanisms of PSF at the transcriptomic and metabolomic levels, laying a theoretical foundation for the broader application of PSF as bio-phosphorus fertilizers in the future.

## 1 Introduction

Phosphorus (P) is a fundamental nutrient for plant growth and development and exists mainly in soil as inorganic and organic forms. The majority of P in soils remains inaccessible to plants (Zeng et al., 2017; Wang et al., 2022a; Lu et al., 2023). Available phosphorus (AP) deficiency affects 74% of China’s arable soil and over 40% of arable soil globally (Roberts and Johnston, 2015; Yu et al., 2019; Ding et al., 2022), creating a high demand for chemical phosphate fertilizers. Using organic or inorganic P fertilizers is crucial for achieving maximum yield but involves high costs (Dário et al., 2018; Zhang et al., 2021a). Additionally, excessive chemical phosphate fertilizer leads to soil nutrition imbalances, heavy metal accumulation, water eutrophication, and environmental pollution (Huang et al., 2017; Park et al., 2021; Ding et al., 2022). Thus, an urgent need exists for an eco-friendly and economically viable approach to ensure a continuous P supply for plants.

Soil hosts a diverse microbiota, with phosphate-solubilizing microbes (PSMs) standing out as beneficial microorganisms capable of converting organic and inorganic insoluble phosphorus compounds into a soluble form easily absorbed by plants (Chawngthu et al., 2020; Wang et al., 2022a; Lu et al., 2023). PSMs enhance plant growth by producing growth-promoting compounds (Ahemad and Kibret, 2014; Rasul et al., 2019) and synthesizing phytohormones vital for plant development. Reducing reliance on chemical phosphorus fertilizers, PSMs play a crucial role in sustainable agricultural practices.

PSMs, diverse in nature, encompass phosphate-solubilizing bacteria (PSB) and phosphate-solubilizing fungi (PSF). The phosphorus solubilization mechanisms involve releasing organic acids, enzymes, and ions such as gluconic acid, phosphatase, and carbonic acid to dissolve insoluble phosphorus (Rawat et al., 2020; Estrada-Bonilla et al., 2021; Nacoon et al., 2021). These mechanisms lower the soil pH, facilitating plant P uptake and usage. Despite numerous studies showcasing PSMs as bio-phosphorus fertilizers to enhance crop yields, a lack of knowledge regarding their mechanisms impedes their further and widespread application (Elhaissoufi et al., 2021). Fortunately, recent advancements in genomics, transcriptomics, metabolomics, and other omics technologies offer robust methods to elucidate the P-solubilizing mechanisms. While multi-omics analyses have unveiled the P-solubilizing mechanism in PSB, specifically, the phosphorus-solubilizing capacity of PSB is likely related to the regulation of genes involved in transport and catabolism, carbohydrate metabolisms, gluconic acid synthesis and other metabolic pathway (Li et al., 2017; Zhang et al., 2020, 2021a; Ding et al., 2022; Lin et al., 2023; Lu et al., 2023). However, studies on the P-solubilizing mechanisms of PSF are still limited.

In a prior study, we identified a PSF strain *Trametes gibbosa* (*T. gibbosa*) T-41 from the soil. T-41 exhibited varying phosphorus solubilization capacities under inorganic (tricalcium phosphate) and organic phosphorus sources (calcium phytin). While the total phenolic contents, antimicrobial activity, and enzyme-producing traits of *T. gibbose* have been documented (Johnsy and Kaviyarasana, 2011; Ma et al., 2013; Zhang et al., 2021b), there are no studies on its P-solubilizing mechanisms. Hence, we (i) explored T-41’s P-solubilizing mechanisms by assessing phosphatase and organic acid production under different phosphorus sources; (ii) investigated the P-solubilizing mechanisms through transcriptomic and metabolomic analyses, and (iii) inferred T-41’s P-solubilization mechanisms under different phosphorus sources via combined the transcriptomic and metabolomic analysis.

## 2 Materials and methods

*T. gibbosa* T-41 isolated from agricultural soil collected in Huidong, Sichuan, China (102°14′40.45″E, 26°39′18.97″N). The strain has been preserved at the China Center for Type Culture Collection under preservation number CCTCC NO: M2022701, and stored at the Laboratory of the Department of Applied Microbiology, Sichuan Agricultural University, Chengdu, China.

### 2.1 Cultivation of T-41 under different phosphorus sources and determination of P solubilization capacity

To prepare the seed liquid medium, the strain was inoculated into PDB liquid culture medium (200 g of potato, 20 g of dextrose, and 1,000 mL of deionized water, with pH 6.8 ~ 7.2) and cultured at 25°C and 180 r/min until reaching the exponential growth stage with an OD_600_ value of 0.6. Subsequently, 5 mL of the seed liquid was transferred to individual 50 mL flasks containing 30 mL of modified Pikovskaya’s liquid media (modified by replacing the original phosphorus source with 5 g/L Ca_3_(PO_4_)_2_ or calcium phytin while retaining other components as per the original recipe). Based on the previous study (Li et al., 2022), two distinct recalcitrant P sources - Ca_3_(PO_4_)_2_ (Ca-P treatment) and phytin (C_6_H_6_Ca_6_O_24_P_6_; Phytin-P treatment) were added at a concentration of 5 g/L. The treatments were conducted in 6 replications. After incubation, soluble P was quantified using the ammonium molybdate spectrophotometric method (WeiXie et al., 2022). At 168 h, the fermentation broth underwent centrifugation at 8,000 r/min for 10 min. One part of the supernatant (approximately 10 mL) was used for determining organic acids and phosphatase activity, while the other was frozen in liquid nitrogen and stored at −80°C. The mycelia were harvested by filtration, washed three times with sterile water, dried with sterile filter papers, instantly frozen using liquid nitrogen, and stored at −80°C.

### 2.2 Measurement of organic acids produced by strain *Trametes gibbosa* T-41

The identification and quantification of organic acids in the T-41 inoculated fermentation broth were conducted using high-performance liquid chromatography. Chromatographic conditions included an Agilent Infinity Lab Poroshell120 SB-C8 column (4.6 mm × 100 mm, 2.7 μm), mobile phase 0.02 mol/L NH_4_H_2_PO_4_-H_3_PO_4_ (pH = 2.9), flow rate 0.4 mL/min, column temperature 30°C, and detection wavelength 210 nm. The organic acid standards used were oxalic acid, citric acid, succinic acid, fumaric acid, tartaric acid, formic acid, acetic acid, gluconic acid, and malic acid (10 mg/mL).

### 2.3 Determination of phosphatase activities of strain *Trametes gibbosa* T-41

The activity of acid and alkaline phosphatase secreted by strain T-41 during incubation was analyzed using the disodium phenyl phosphate method (DPP) (Kelly et al., 2019). Briefly, 1 mL fermentation broth was incubated with 4 mL universal buffer (pH = 6.5 for acid phosphatase and pH = 11 for alkaline phosphatase) and 1 mL 25 mM DPP for 1 h at 25°C. Subsequently, 1 mL 0.5 M CaCl_2_ and 4 mL 0.5 M NaOH were added to terminate the reaction. The concentration of DPP was measured spectrophotometrically based on absorbance at 510 nm.

### 2.4 Transcriptomic analysis and untargeted LC–MS metabolomic analysis

For the transcriptomic analysis, total RNA was extracted from the mycelia using a pre-chilled TRIzol reagent following the manufacturer’s instructions (Invitrogen, Carlsbad, United States). The quantity and quality of the total RNA were assessed using NanoDrop 2000 (Thermo Scientific) and the Agilent Bioanalyzer 2,100 system, respectively. RNA samples with integrity number values above 8.0 were employed to construct the sequencing library. Subsequently, paired-end sequencing was performed on Illumina NovaSeq at Personal Biotechnology Co., Ltd. (Shanghai, China). To ensure high-quality clean reads, raw reads were trimmed and quality-controlled using Seqprep software with default parameters. Evaluation of Q20, Q30, and sequence replication levels was conducted. Following the removal of adapters, poly-N, and low-quality value (QV < 20) reads, high-quality clean reads were *de novo* assembled using Trinity software (Garber et al., 2011). Differential analysis of gene expression was performed using DESeq, genes with |log2FoldChange| > 1 and *p*-value<0.05 were considered differentially expressed genes (DEGs; Love et al., 2014). The Gene Ontology (GO) database was used to compile the functional analysis of DEGs in terms of molecular functions (MF), biological processes (BP), and cellular components (CC). The Kyoto Encyclopedia of Genes and Genomes (KEGG) was used to investigate the signaling pathways of DEGs. The GO and KEGG pathway analyses of the DEGs were performed using the cluster Profiler package in R (*p* value cutoff = 0.05; Zhu et al., 2021; R Core Team, 2023).

Metabolomic analysis was performed on a liquid chromatography-mass spectrometry (LC–MS) system at Personal Biotechnology Co., Ltd. (Shanghai, China). MS raw data files were converted to mzXML format using Proteowizard software. Subsequent data management encompassed raw peak identification, peaks filtration, peaks alignment, and internal standard normalization processed via the R package XCMS v3.3.2 (Wang et al., 2022b). Metabolites with a VIP > 1 and *p*-values (< 0.05) derived from Student’s t-test (T-test) on normalized peak areas were considered differentially accumulated metabolites (DAMs). Metabolites were identified and annotated using HMDB (Wishart et al., 2007), Metlin (Smith et al., 2005), Massbank (Horai et al., 2010), LipidMaps (Fahy et al., 2007), Mzclound (HighChem, 2016), and an in-house MS/MS database (Personal Biotechnology Co., Ltd., Shanghai, China; Cai et al., 2023). Metabolic pathways analysis of identified metabolites was performed using KEGG and MetaboAnalyst 3.0 (Xia et al., 2015).

### 2.5 Integrative analysis of transcriptomic and metabolomic

The DEGs and DAMs were mapped to the KEGG pathway database to acquire common pathway information (Ma et al., 2023). Pearson correlations between the DEGs and DAMs were calculated in R. All integrated analyses were conducted by Shanghai Personal Biotechnology Co., Ltd. (Shanghai, China). Nine-quadrant plots illustrated the differential multiplicity of substances with Pearson correlation coefficients above 0.80 and *p*-values less than 0.05 in each subgroup.

### 2.6 qRT-PCR validation of RNA-Seq data

Eight differentially expressed genes were selected for validation by quantitative real-time PCR (qRT-PCR). qRT-PCR was performed on a CFX96 Real-Time System (BIO-RAD) with SYBR green as the fluorescent dye according to the manufacturer’s protocol. qRT-PCR validation included three biological replicates with three technical replicates and GAPDH as the internal control gene as previously described (Huggett et al., 2005). The primers applied are in Supplementary Table S1. Genes were considered differentially expressed when fold change (FC) ≥ 1.5 or ≤ 0.667 and *p* < 0.05.

### 2.7 Statistical analyses

Differences in the transcriptomes and metabolomes were visualized using principal co-ordinates analysis (PCoA) and tested with permutational multivariate analysis of variance (PERMANOVA) and pairwise PERMANOVA with 999 permutations in R packages vegan v2.6.4 (Oksanen et al., 2022). Figures were created using Origin 9.0 software and Genescloud.^1^ The figures were merged using Adobe Photoshop 2020. One-way ANOVA and independent t-test were carried out using IBM SPSS version 22.0 software (Chicago, IL, United States) for statistical analysis.

## 3 Results

### 3.1 Phosphorus solubilizing characteristics of *Trametes gibbosa* T-41

**Figure 1:**
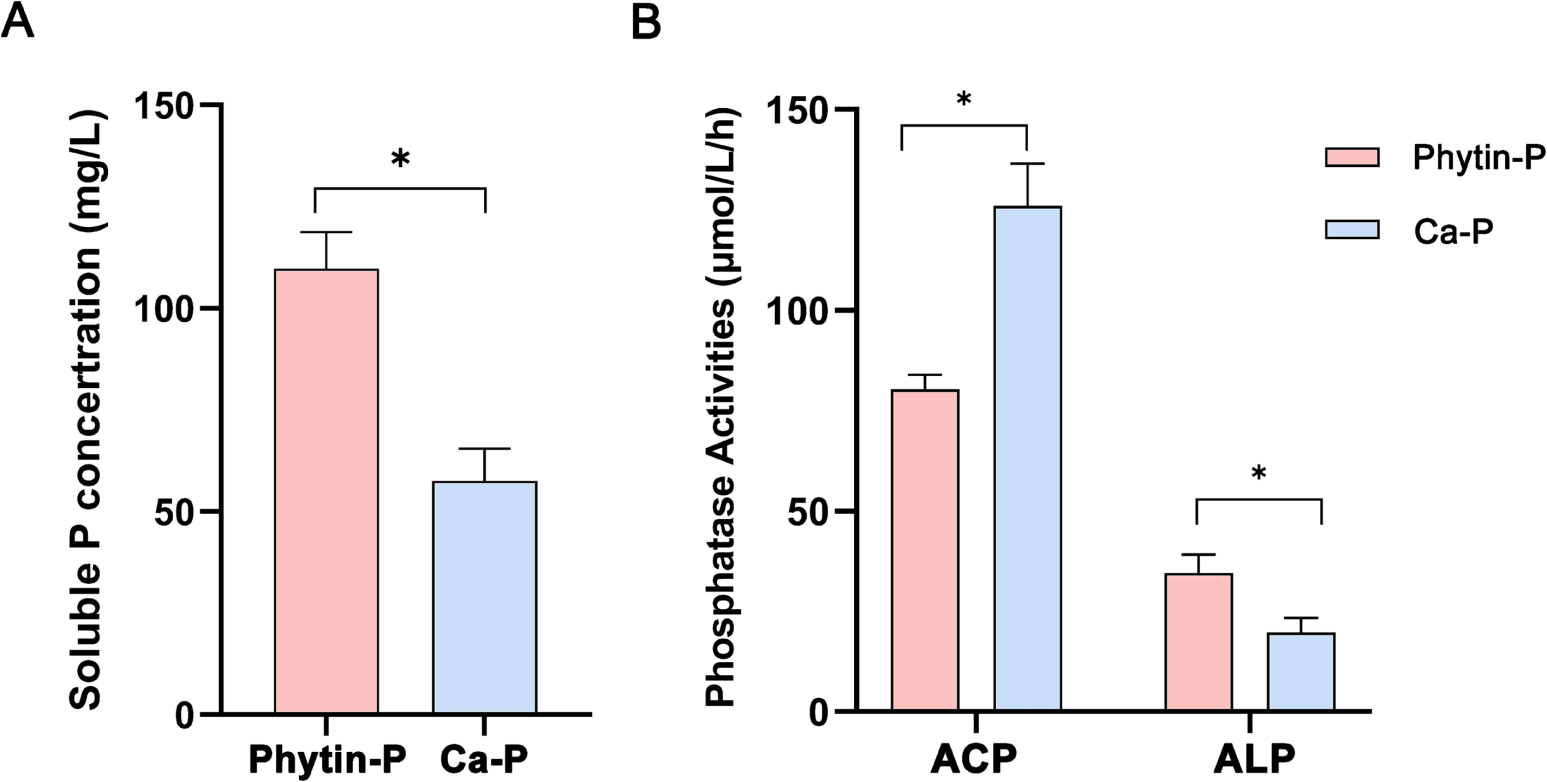
Soluble P concentration **(A)** and phosphatase activity **(B)** of *Trametes gibbosa* T-41 under two P sources. Differences in soluble P concentration and phosphatase activities between the two treatments were compared by T-test. *indicates a statistically significant difference at *p* < 0.05.

After incubation in the PDB medium for 168 h, the content of soluble phosphorus was higher in the Phytin-P treatment than in the Ca-P treatment (Figure 1A; *p <* 0.05). Acid phosphatase (ACP) activity was lower and alkaline phosphatase (ALP) activity was higher in the Phytin-P treatment than in the Ca-P treatment (*p <* 0.05; Figure 1B). The morphology and biomass of T-41 under different phosphorus sources were showed in Supplementary Figure S1.

A total of 12 organic acids were detected (Table 1). Compared with the Ca-P treatment, the content of oxalic acid, tartaric acid, formic acid, malonic acid, maleic acid, and succinic acid was higher in the Phytin-P treatment (*p* < 0.05; Table 1).

**Table 1:** The organic acid contents in *Trametes gibbosa* T-41 cultures with different phosphorus sources.

Organic acid (ng/μL)	Oxalic acid	Tartaric acid	Formic acid	Malic acid	Malonic acid	α-ketoglutaric acid
Phytin-P	1.080 ± 0.015^a^	0.008 ± 0.01^a^	0.107 ± 0.005^a^	0.755 ± 0.056^b^	0.427 ± 0.094^a^	0.005 ± 0.001^a^
Ca-P	0.338 ± 0.048^b^	0.002 ± 0.001^b^	0.070 ± 0.005^b^	5.102 ± 1.231^a^	0.116 ± 0.109^b^	0.005 ± 0.006^a^
Organic acid (ng/μL)	Lactic acid	Acetic acid	Citric acid	Maleic acid	Fumaric acid	Succinic acid
Phytin-P	0.447 ± 0.065^b^	1.11 ± 0.011^b^	0.001 ± 0.000^a^	3.42 ± 4.047^a^	0.001 ± 0.000^a^	2.67 ± 0.320^a^
Ca-P	1.69 ± 0.009^a^	1.66 ± 0.019^a^	0.002 ± 0.000^a^	0.251 ± 0.019^b^	0.002 ± 0.000^a^	0.022 ± 0.003^b^

Data are mean ± SE (*n* = 3). Different superscript letters in a column indicate a statistically significant difference between treatments (T-test, *p* < 0.05).

### 3.2 Effects of phosphorus source on the gene expression profile

**Figure 2:**
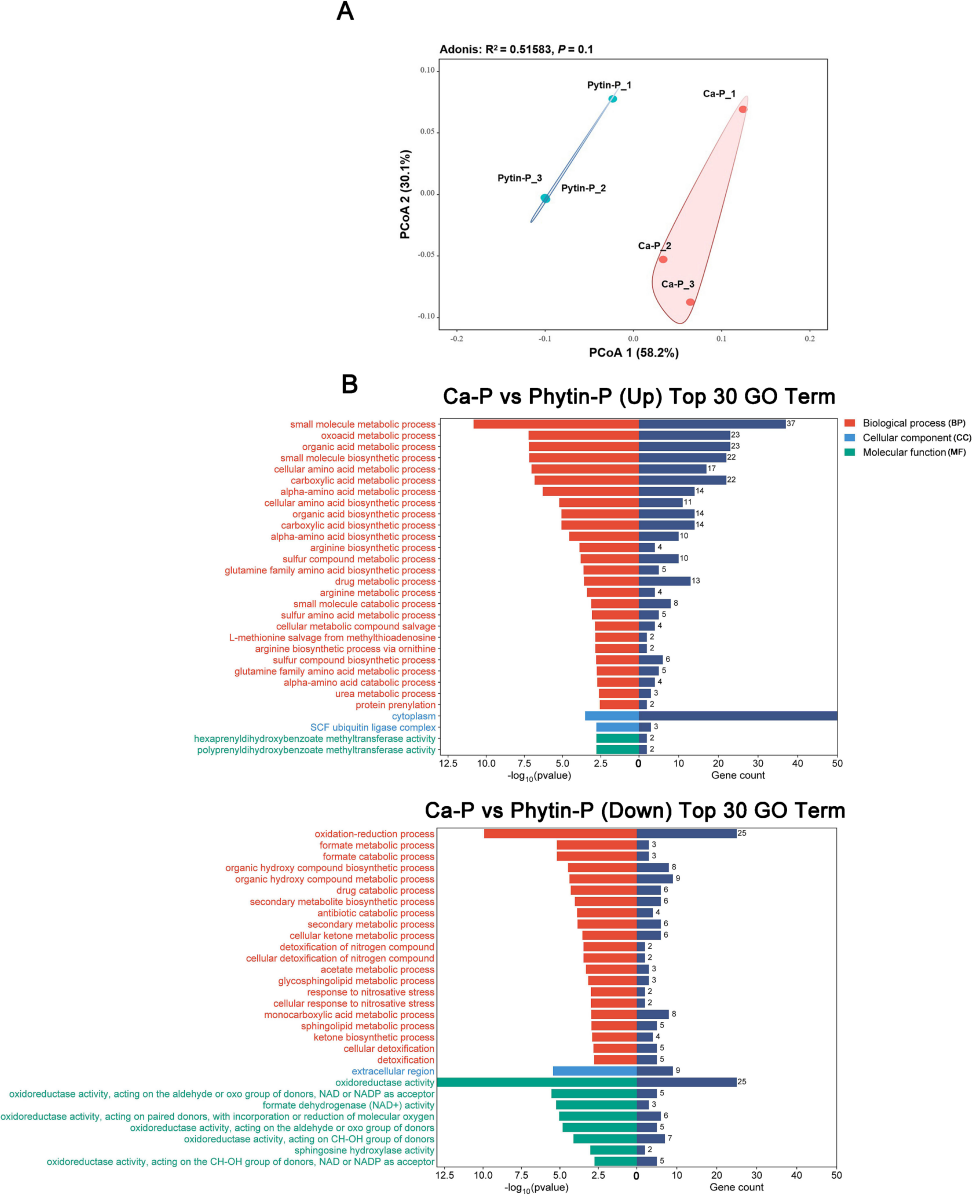
Transcriptional profiling of *T. gibbosa* T-41 under Ca-p and Phytin-P treatment. PCoA ordination of the transcriptomes **(A)**. The top 30 enriched GO annotations in the DEGs are up-regulated and down-regulated in Ca-P vs. Phytin-P as the phosphorus source **(B)**.

Despite the clear separation of the transcriptomes from the Phytin-P and Ca-P treatments in the principal component analysis (PCoA; Figure 2A), the overall difference in gene expression was not statistically significant (PerMANOVA, *p* > 0.05; Supplementary Table S2). A total of 350 genes were identified as differentially expressed genes (DEGs); compared with the Ca-P treatment, 179 genes were up-regulated and 171 down-regulated in the Phytin-P treatment. The qRT-PCR gene expression of eight selected DEGs agreed with the differential expression observed in RNA-Seq analysis (Supplementary Table S3).

To assess the biological functions of DEGs due to different phosphorus sources, GO enrichment analysis was performed on DEGs. In the BP aspect, Phytin-P up-regulated DEGs were enriched in various processes such as organic acid biosynthesis and amino acid metabolism, while most down-regulated DEGs were linked to oxidation–reduction and drug metabolic processes (Figure 2). In the CC aspect, Phytin-P up-regulated DEGs were enriched in the cytoplasm and SCF ubiquitin ligase complex, whereas down-regulated DEGs were enriched in the extracellular region. In the MF aspect, Phytin-P up-regulated DEGs were enriched in activities like hexaprenyldihydroxybenzoate methyltransferase and polyprenyldihydroxybenzoate methyltransferase, while down-regulated DEGs were associated with oxidoreductase activity.

### 3.3 Effects of phosphorus source on the metabolite profile

**Figure 3:**
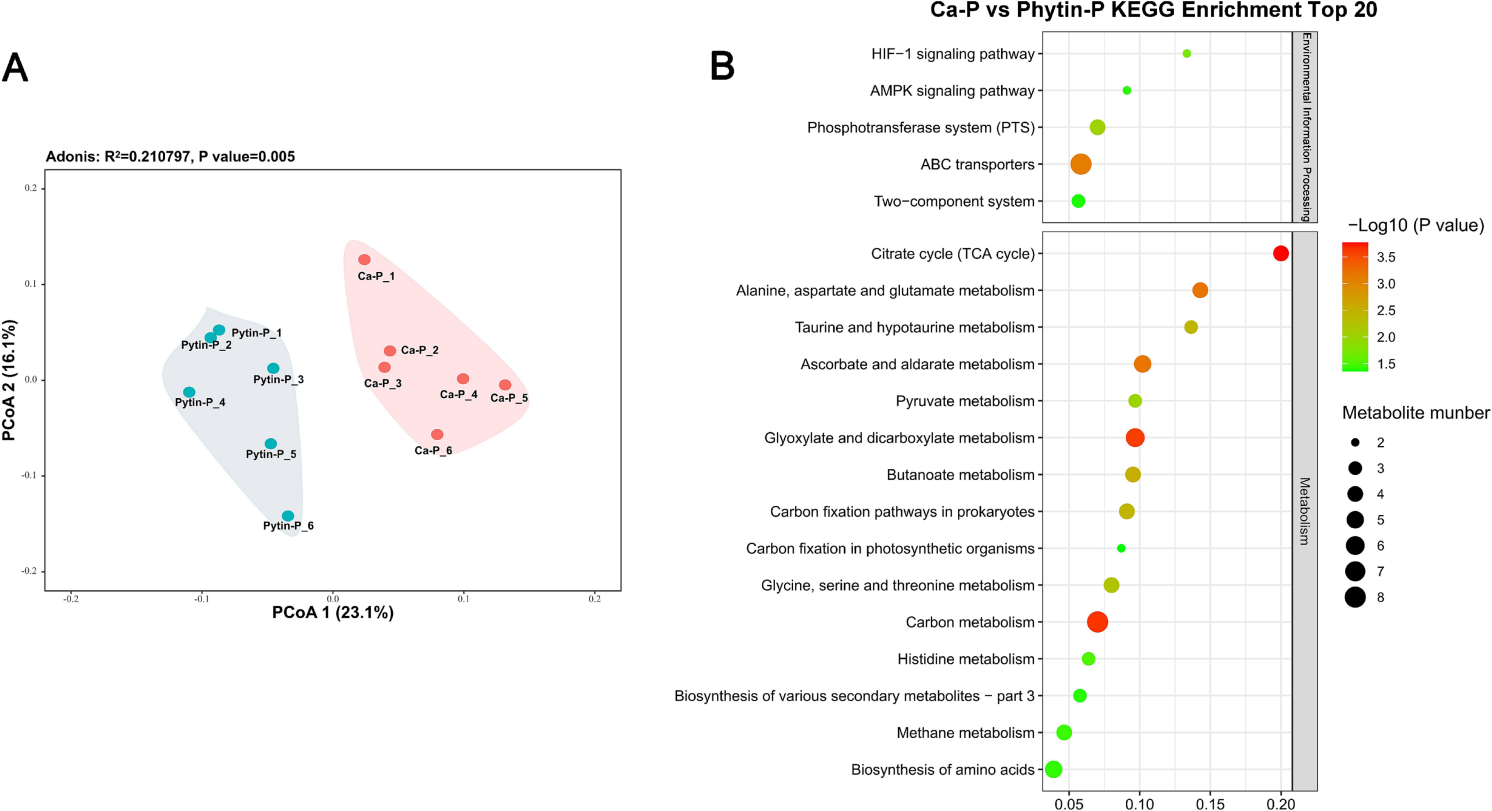
Metabolic profiling of *T. gibbosa* T-41 under Ca-p and Phytin-P treatment. PCoA ordination of the metabolomes **(A)**. The top 20 enriched KEGG pathways in the DAMs in Ca-P vs. Phytin-P as the phosphorus source **(B)**.

PCoA (Figure 3A) demonstrated distinct metabolite patterns, implying alterations in metabolite abundances in *T. gibbosa* T-41 due to different phosphorus sources (PerMANOVA *p <* 0.05). A total of 114 metabolites were identified as differentially accumulated metabolites (DAMs), encompassing 67 up-regulated and 47 down-regulated metabolites in Ca-P vs. Phytin-P. Notably, the up-regulated DAMs in Ca-P vs. Phytin-P treatments outnumbered the down-regulated DAMs, consistent with observations in DAMs with the highest concentrations (Table 2).

**Table 2:** Key metabolites in *T. gibbosa* T-41 cultures and their fold changes in Ca-P vs. Phytin-P as the phosphorus source.

Metabolite name	Metabolite name (Subclass)	log_2_(FC)	Significance
Benzenoids	Benzenediols	−0.20	***
	Halophenols	−0.52	***
	Biphenyls and derivatives	0.58	***
	Biphenyls and derivatives	−0.72	*
Lignans, neolignans and related compounds	Podophyllotoxins	−0.39	***
Lipids and lipid-like molecules	Hydroxysteroids	−0.60	***
	Estrane steroids	0.43	***
	Sulfated steroids	1.51	***
Nucleosides, nucleotides, and analogs	Purine 2′-deoxyribonucleosides	0.29	***
Organic acids and derivatives	Amino acids, peptides, and analog	0.40	***
	Amino acids, peptides, and analogs	−0.29	***
	Gamma-keto acids and derivatives	0.21	***
	Alpha-keto acids and derivatives	−1.20	***
	Amino acids, peptides, and analogs	0.77	***
	Amino acids, peptides, and analogs	1.05	***
	Beta hydroxy acids and derivatives	0.34	*
	Tricarboxylic acids and derivatives	−0.42	*
Organic oxygen compounds	Carbohydrates and carbohydrate conjugates	0.34	***
	Carbohydrates and carbohydrate conjugates	−0.24	***
	Carbohydrates and carbohydrate conjugates	−0.60	***
	Carbohydrates and carbohydrate conjugates	0.45	***
	Carbonyl compounds	1.38	***
	Carbohydrates and carbohydrate conjugates	−0.62	***
	Carbohydrates and carbohydrate conjugates	−0.62	***
	Carbohydrates and carbohydrate conjugates	−0.82	**
	Carbohydrates and carbohydrate conjugates	0.22	**
	Carbohydrates and carbohydrate conjugates	0.79	*
Organoheterocyclic compounds	Pyranones and derivatives	1.43	***
	Imidazolines	0.63	***
	Purines and purine derivatives	0.73	***
	Pyrimidines and pyrimidine derivatives	−0.32	***
	1-benzopyrans	−0.58	***
	Indolyl carboxylic acids and derivatives	−0.59	*
Phenylpropanoids and polyketides	Flavones	0.81	***
	Linear diarylheptanoids	0.99	**

*p < 0.05, **p < 0.01, and ***p < 0.001; *n* = 6.

All DAMs with KEGG ID were subjected to pathway enrichment analysis in the KEGG database to elucidate their relationships. Most DAMs were enriched in the TCA cycle and others were associated with amino acid metabolism pathways and carbon metabolism (Figure 3B). Notably, the majority of key metabolites belonged to organic oxygen compounds and organic acids and derivatives. Most organic acids and derivatives were up-regulated in Phytin-P vs. Ca-P, in agreement with the higher organic acid production observed under Phytin-P treatment.

### 3.4 Integration of transcriptomic and metabolomic datasets

**Figure 4:**
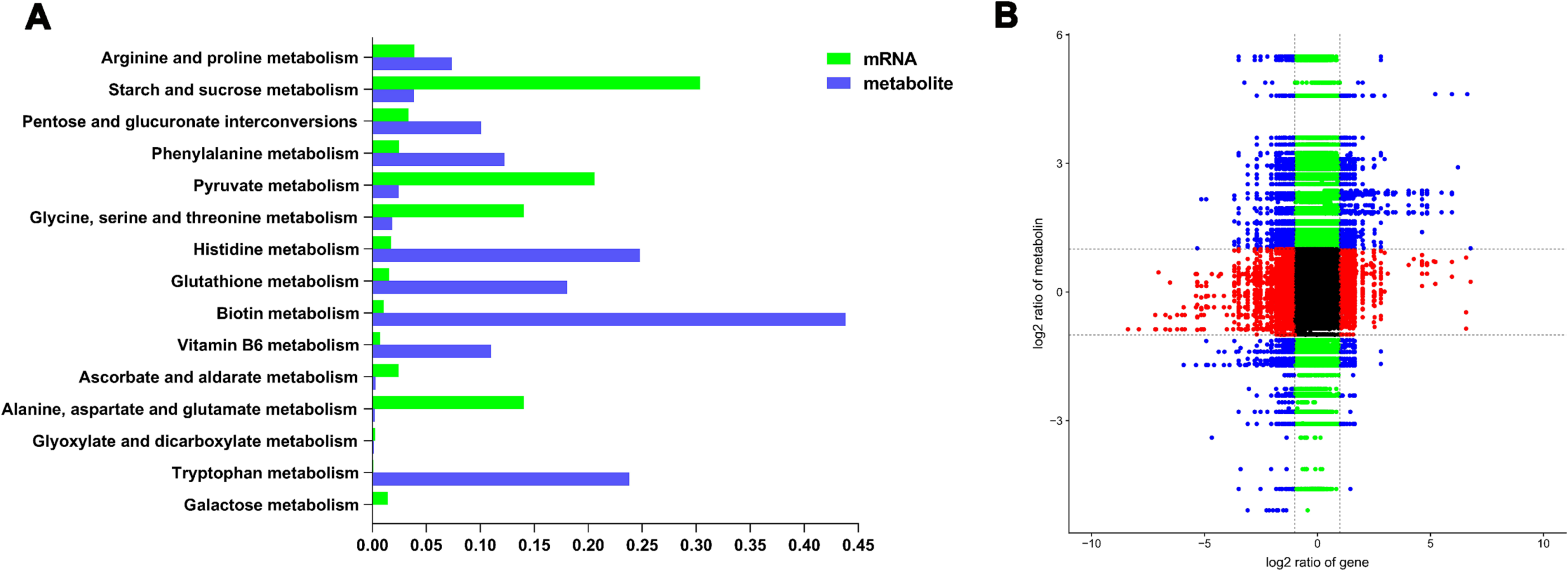
Integrated analysis of the transcriptomic and metabolomic of *T. gibbosa* T-41 under Phytin-P treatment. The top 10 metabolism pathways from KEGG level 2 enrichment analysis **(A)**. Correlation between DEGs and DAMs **(B)**.

Correlation analysis revealed a connection between DEGs and DAMs (Supplementary Figure S2). By merging the transcriptomic and metabolomic analysis datasets, we identified common enrichment KEGG Level 2 pathways linked to metabolism (Figure 4A). Two hundred genes and metabolites correlated significantly, including 110 positive and 90 negative correlations (Figure 4B).

**Figure 5:**
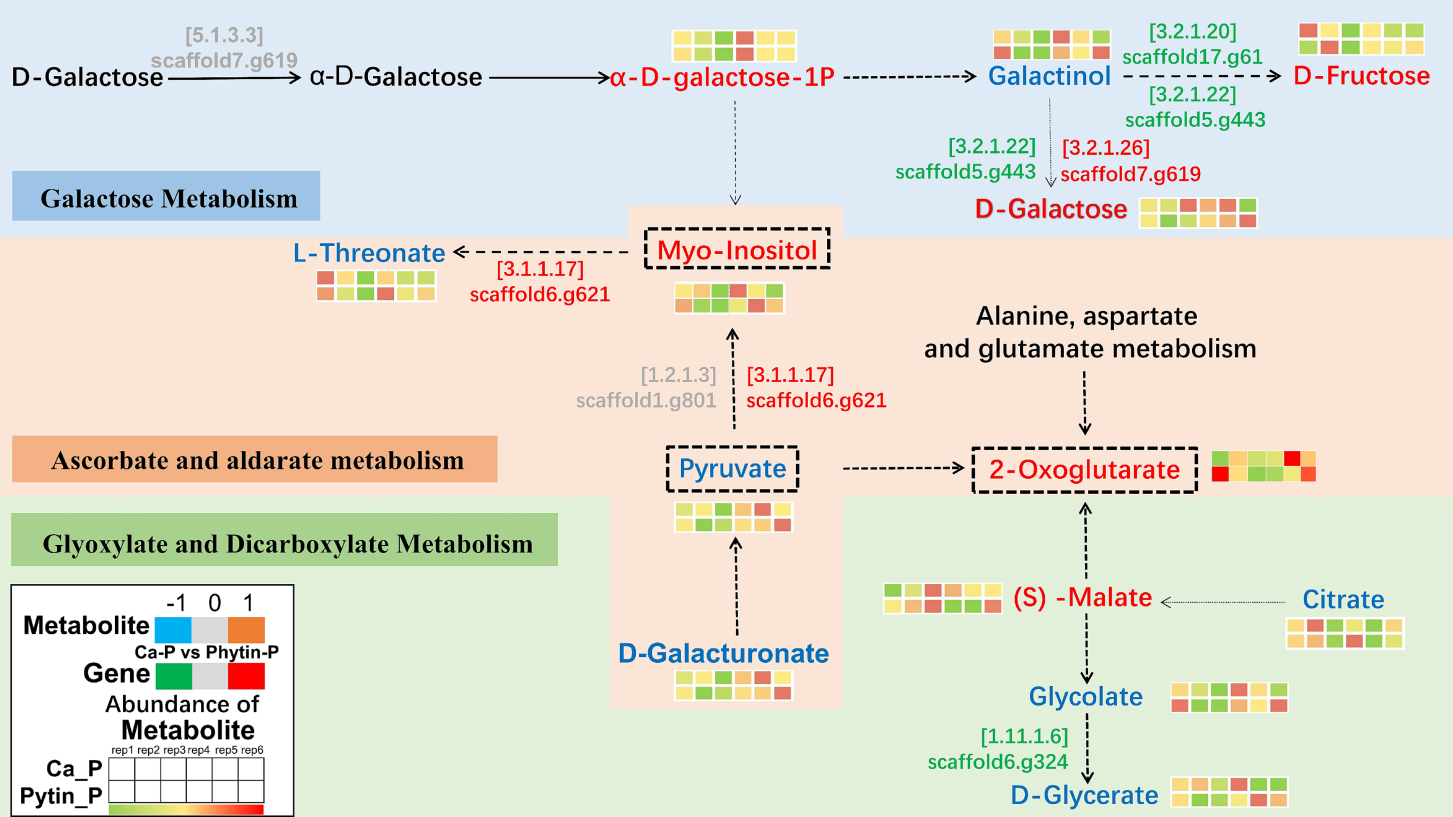
Genes and metabolites in Galactose metabolism, Glyoxylate and dicarboxylate metabolism, Ascorbate and aldarate metabolism pathways. The colored text indicates substances detected in this study. For metabolites, red font indicates up-regulation, blue font indicates down-regulation and gray font indicates insignificant change. For genes, green font indicates down-regulation. The EC number of the enzyme encoded by a gene is shown above the gene identifier.

To delve deeper into how different phosphorus sources impact *T. gibbosa* T-41 functioning, relevant genes and metabolites were selected to map the pathway based on the KEGG pathway database and the transcriptomic and metabolomic co-enrichment pathway results (Figure 5). The results showed that joint-enriched pathways were related to galactose metabolism, glyoxylate and dicarboxylate metabolism, and ascorbate and aldarate metabolism. Common metabolites from these pathways, namely myo-inositol, pyruvate, and 2-oxoglutarate, exhibited changes (Table 3). These compounds are presumed to play a pivotal role in enhancing the phosphorus solubilization capacity of T-41. Specifically, the up-regulation of the gene scaffold6.g621 potentially increases myo-inositol expression, thereby enhancing T-41 phosphorus solubilization ability.

**Table 3:** Metabolites in the galactose metabolism, glyoxylate and dicarboxylate metabolism pathway in *T. gibbosa* T-41 cultures and their fold changes in Ca-P vs. Phytin-P as the phosphorus source.

Pathway	Metabolite	log_2_(FC)	Significance
Galactose Metabolism	α-D-galactose-1P	0.22	**
	Galactinol	−0.62	***
	D-Fructose	0.45	***
Ascorbate and aldarate metabolism	L-Threonate	−0.82	**
	D-Galacturonate	−1.81	***
Glyoxylate and Dicarboxylate Metabolism	(S) -Malate	0.34	*
	Glycolate	−2.49	**
	D-Glycerate	−0.12	***
	Citrate	−0.42	*
Ascorbate and aldarate metabolism/Galactose Metabolism	Myo-inositol	2.41	***
Ascorbate and aldarate metabolism/Glyoxylate and Dicarboxylate Metabolism	Pyruvate	−1.20	***
	2-Oxoglutarate	1.56	**

*p < 0.05, **p < 0.01, and ***p < 0.001; *n* = 6.

## 4 Discussion

### 4.1 Phosphorus solubilizing properties of *Trametes gibbosa* T41

Phosphorus is an essential nutrient for all life forms. Various microorganisms known as PSMs can solubilize soil phosphate. In recent years, a variety of PSF have been identified, including *Penicillum* sp. PK112, T*richoderma harzianum* OMG08, *Aspergillus aculeatus* P93, *Penicillium daleae,* and other endophytic symbionts such as arbuscular mycorrhizal (AM) fungi (Adhikari and Pandey, 2019; Mercl et al., 2020; Kumar et al., 2020; Hao et al., 2020). PSF plays a crucial role in plant growth and development by converting unavailable phosphate forms into soluble form, primarily through the secretion of organic acids and phosphatase enzymes, particularly acid phosphatase (An et al., 2021; Lalmuankimi and Ralte, 2021; Kang et al., 2021). Phosphatases help release inorganic phosphate from organically bound phosphate, contributing to the enrichment of soluble P (Jyothi and Thippeswamy, 2023). We isolated a PSF *Trametes gibbosa* T-41 from the soil. *T. gibbosa* T-41 exhibited higher efficiency in dissolving phosphorus from organic phosphorus source (Phytin-P) than inorganic phosphorus source (Ca-P). Compared with other studies, T-41 had a high phosphorus solubilizing capacity under Phytin treatment (Jiang et al., 2020; Kumar et al., 2020). To assess the phosphorus solubilization mechanisms of *T. gibbosa* T-41, we examined phosphatase and organic acid production of *T. gibbosa* T-41 under varying phosphorus source culture conditions, revealing distinct results depending on the phosphorus sources. ACP and ALP activities differed between organic and inorganic phosphorus sources. In line with earlier studies showing large differences in organic acid types and yields in PSF under different culture conditions (Li et al., 2016; Jiang et al., 2020), *T. gibbosa* T-41 produced more organic acids in the Phytin-P treatment.

Previous studies have indicated that phosphatase production in PSMs is closely linked to strain characteristics and culture conditions (Fitriatin et al., 2020; Lalmuankimi and Ralte, 2021; Jyothi and Thippeswamy, 2023). The capability of phosphate-solubilizing microbes to dissolve various phosphate forms depends on the organic compounds they produce and the phosphate source (Serna-Posso et al., 2017; Fitriatin et al., 2022; Wang et al., 2022a). Organic acids like citric, oxalic, lactic, and tartaric acids contribute to dissolving phosphate minerals by supplying protons and forming organic acid anion metal complexes (Pande et al., 2017; Yadav et al., 2017; Fitriatin et al., 2020). Environmental conditions can influence the ability to secrete organic acids (Li et al., 2016; Fitriatin et al., 2020). In this study, oxalic and maleic acids were identified as major contributors to phosphorus solubilization under Phytin-P treatment, aligning with their proton-donating and complexation properties. To the best of our knowledge, this is the first time that the P solubilization mechanisms of a PSF have been studied. The results suggest the potential involvement of phosphatases and organic acid secretion. However, unveiling the specific mechanisms behind *T. gibbosa* T-41 phosphorus solubilization in different phosphorus sources necessitates further transcriptomic and metabolomic analyses.

### 4.2 Phosphorus solubilizing related DEGs and DAMs

Analyzing the phosphorus solubilization and organic acid secretion by *T. gibbosa* T-41 through transcriptomic and metabolomic analysis revealed that different phosphorus sources altered the gene expression profile. Compared to Ca-P treatment, more DEGs were up-regulated with Phytin-P as the P source, indicating that Phytin-P may stimulate the gene expression to improve phosphate solubilization. Moreover, GO analysis showed that most DEGs were associated with the BP process. Among them, more up-regulated DEGs were enriched in various organic acid biosynthesis and amino acid metabolism pathways. The acidolysis pathway is considered to be a major aspect of the phosphate-solubilizing mechanism; many organic acids participate in microbial solubilization of insoluble inorganic P (Ludueña et al., 2018; Zhang et al., 2021a; Wang et al., 2022a; Yu et al., 2021). Correspondingly, the down-regulated DEGs related to MF were the most abundant, and more down-regulated DEGs were involved in oxidation–reduction and formate metabolic processes. In general, these results showed that promoting the gene expression in organic acid metabolic pathways and repressing the gene expression involved in the formate metabolic process might crucially impact T-41’s phosphorus solubilization under distinct phosphorus sources.

Metabolomic analyses were employed to delve deeper into the mechanisms. The KEGG analysis revealed that most DAMs were enriched in the TCA cycle and alanine, aspartate and glutamate metabolism pathways, while others were found in amino acid metabolism and carbohydrate metabolism pathways. In a study by Lin et al. (2023), research on a PSB *Bacillus megaterium* strain with growth-promoting effects found that most DEGs were involved in energy metabolism, carbohydrate metabolism, and amino acid metabolism compared to the control. Similarly, Wang et al. (2022a) used transcriptomics to analyze DEG expression patterns in the phosphate-solubilizing bacterium W134 under different phosphorus concentrations. Their results suggested the up-regulation of genes in the TCA cycle pathway, which is closely linked to organic acid production. These findings align with our study, indicating that DEGs may be linked to *T. gibbosa* T-41’s response to diverse phosphorus sources, self-regulation of metabolism, and the synthesis and secretion of organic acids. Most low-molecular-weight organic acids are presumably derived from the TCA cycle, which can generate various organic acids like citric acid, *α*-ketoglutaric acid, succinic acid, and malic acid (Vuoristo et al., 2016; Li et al., 2016; Huang et al., 2022). This might explain the variations in organic acid content across different phosphorus sources. Most key metabolites belong to organic oxygen compounds and organic acids and derivatives. The increased abundance of most organic acids and derivatives aligns with the Phytin-P treatment compared with Ca-P.

Several studies have investigated the molecular mechanisms of PSMs by using transcriptome or metabolomics, but most of them are related to bacteria (Zhang et al., 2020; Zhang et al., 2021a; Lin et al., 2023). There are fewer explorations on the phosphorus solubilization mechanism of PSF. We found that when *T. gibbosa* T-41 was under Phytin-P cultivation, the DEGs related to organic acid biosynthesis were up-regulated and most DAMs were enriched in the TCA cycle, leading to the secretion of more organic acids such as oxalic acid, maleic acid and succinic acid.

### 4.3 Combined transcriptomic and metabolomic reveal the P-solubilizing mechanism of *Trametes gibbosa* T41 under Phytin-P

In our study, we observed variations in the phosphorus solubilization mechanism of T-41 across different phosphorus sources. By combining the transcriptomic and metabolomic analysis datasets, the common altered KEGG pathways associated with metabolism were identified. These included galactose metabolism, glyoxylate and dicarboxylate metabolism and ascorbate and aldarate metabolism pathways, and key metabolites linking these pathways, such as myo-inositol, pyruvate, and 2-oxoglutarate, were notably altered. There was substantial down-regulation of genes related to galactose metabolism and glyoxylate and dicarboxylate metabolism pathway, indicating that *T. gibbosa* T-41 may alter its energy production and conversion pathways during phosphorus solubilization (Strijbis and Distel, 2010; Iskandar et al., 2019). Furthermore, in the ascorbate and aldarate metabolic pathway, different phosphorus sources affected the fluxes through the highly expressed myo-inositol enzyme, which transforms inositol into glucuronic acid using oxygen. 2-oxoglutarate was the key intermediate for the TCA cycle (Lancien et al., 2000). T-41 affects compound synthesis by regulating key genes in the TCA cycle to act as a phosphorus solvent.

Multi-omics analysis can be an effective tool for understanding the phosphorus solubilization mechanism of PSMs. Ding et al. (2022) used transcriptomic and metabolomic to reveal the efficient phosphate-solubilizing mechanism of PSB-FP12. Similar to our results, FP12 upregulated the expression of genes involved in the organic acid synthesis pathway and secreted more organic acids. Metabolites such as organic acids play a crucial role in the PSM’s function as a phosphorus solubilizer. Notably, the expression patterns of related genes and organic acid secretion in *T. gibbosa* T-41 differed from those reported in some other PSMs, implying diverse phosphorus-solubilizing mechanisms among various PSMs. It is essential to highlight the relatively limited research on phosphorus solubilization mechanisms in PSF. Our findings offer new insights into unraveling the phosphorus solubilization mechanisms in PSF.

## 5 Conclusion

The PSF strain *Trametes gibbosa* T-41 exhibited varying phosphorus solubilization capacities under organic and inorganic phosphorus sources, with the highest capacity observed in the organic phosphorus source (Phytin-P treatment). The alterations in galactose metabolism, glyoxylate and dicarboxylate metabolism, and ascorbate and aldarate metabolism pathways may primarily account for the enhanced phosphorus solubilization in organic phosphorus sources. The results of this study contribute to a better understanding of phosphorus solubilization in PSF. Future investigations employing genetic and molecular biological methods are crucial to elucidate the detailed mechanisms involved in inducing the phosphorus solubilization mechanism.

## Data Availability

The original contributions presented in the study are publicly available. This data can be found at: https://www.ncbi.nlm.nih.gov/, accession number ON597379; Chinese Typical Cultures Depository Center (CCTCC), depository number CCTCC NO: M2022701.

## Footnotes

^1^ www.genescloud.cn/home
