# Supplementary material for: Transcriptomic and metabolomic analysis of recalcitrant phosphorus solubilization mechanisms in Trametes gibbosa
Source: Front Microbiol. 2025 Feb 4;16:1520459. doi: 10.3389/fmicb.2025.1520459 (PMC11832667; doi:10.3389/fmicb.2025.1520459)
Supplement: Supplementary file 1 [file Data_Sheet_1.docx]

**Supplementary Material**

Table S1. Primer sequences and information of gene for the qRT-PCR in this study

| Gene symbols | Primer sequences (5′ to 3′)’ |
| --- | --- |
| scaffold6.g365 | F: TATTGCTGATACCCTCCT |
|  | R: AGAGTAGAGACGGATGAG |
| scaffold14.g208 | F: AGAGCAAGTTCACTGTTA |
|  | R: ATCCTCAAAGGTCTTCAT |
| scaffold8.g515 | F: GAAGAACACTATCCTCAAGAAG |
|  | R: TTGTATTCGCTCTCGTAGAT |
| scaffold9.g524 | F: GAGTGGTTTCGCATCTAC |
|  | R: CGTGGCATACTTCTTGTT |
| scaffold7.g415 | F: GAATATCCTCACACTACC |
|  | R: AAGTCTTGTTATTGAAGTC |
| scaffold3.g582 | F: AGTTCATCCTCACGCATC |
|  | R: CATGGAAGTACATCGAGTTG |
| scaffold9.g161 | F: AAGATACCAAGGTGCTCTG |
|  | R: CGTACTCAATCGCCTCTT |
| scaffold7.g101 | F: GCCTTCCATTCGTTGAAC |
|  | R: AGAACAGCATAGCCAGAG |
| ITS | F: GGAAGTAAAAGTCGTAACAAGG |
|  | R: GCTGCGTTCTTCATCGATGC |

Table S2. PerMANOVA tests between Ca-P and Phytin-P genes

| Category | Paris | R^2^ | P value |
| --- | --- | --- | --- |
| genes | Ca-P VS Pytin-P | 0.516 | 0.1 |
| metabolites | Ca-P VS Pytin-P | 0.208 | 0.05 |

Table S3 |log2FoldChange| of qRT-PCR gene

| Gene id | qRT-PCR  Fold Change  (Ca_P/Pytin_P) | RNAseq  Fold Change  (Ca_P/Pytin_P) |
| --- | --- | --- |
| scaffold6.g365 | 2.74 | 1.59 |
| scaffold7.g101 | -2.48 | -1.53 |
| scaffold8.g515 | -2.08 | -1.36 |
| scaffold9.g161 | 1.95 | 1.16 |
| scaffold14.g208 | -3.57 | -1.96 |
| scaffold3.g582 | 2.27 | 1.68 |
| scaffold7.g415 | 2.19 | 1.17 |
| scaffold9.g524 | 2.90 | 1.88 |

Data were analyzed using t-tests, with different numbers indicating significant differences (P<0.05)

**
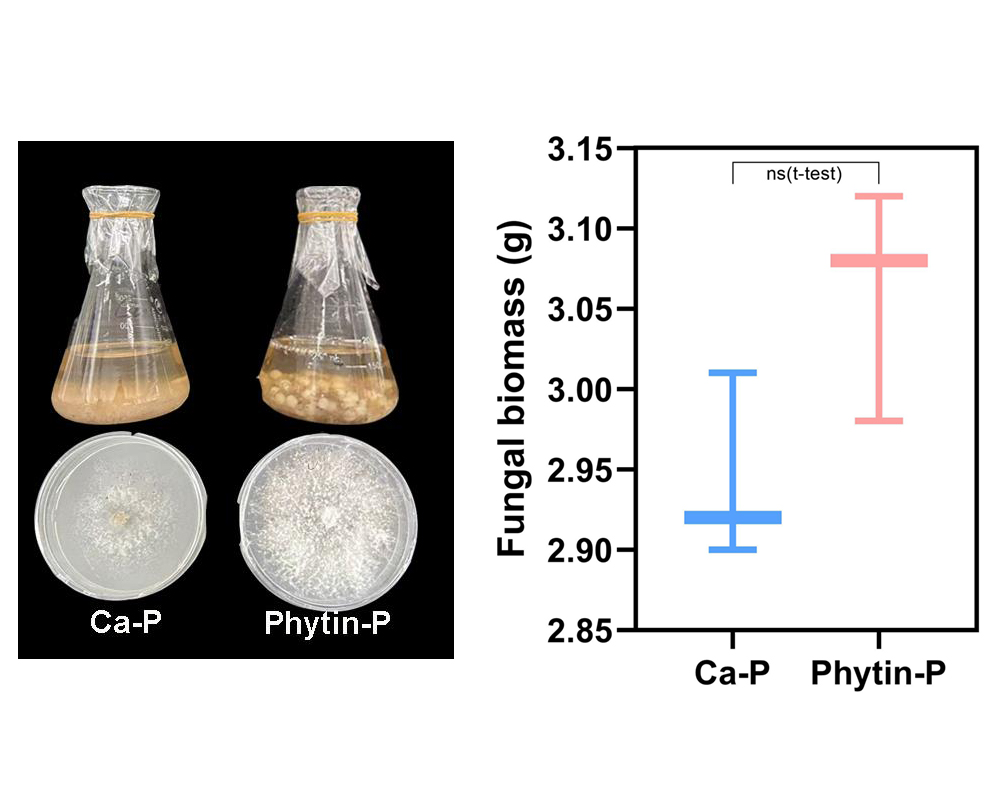
**

Fig. S1 Morphology and biomass of T-41 under different phosphorus sources (incubated for 168 hours)


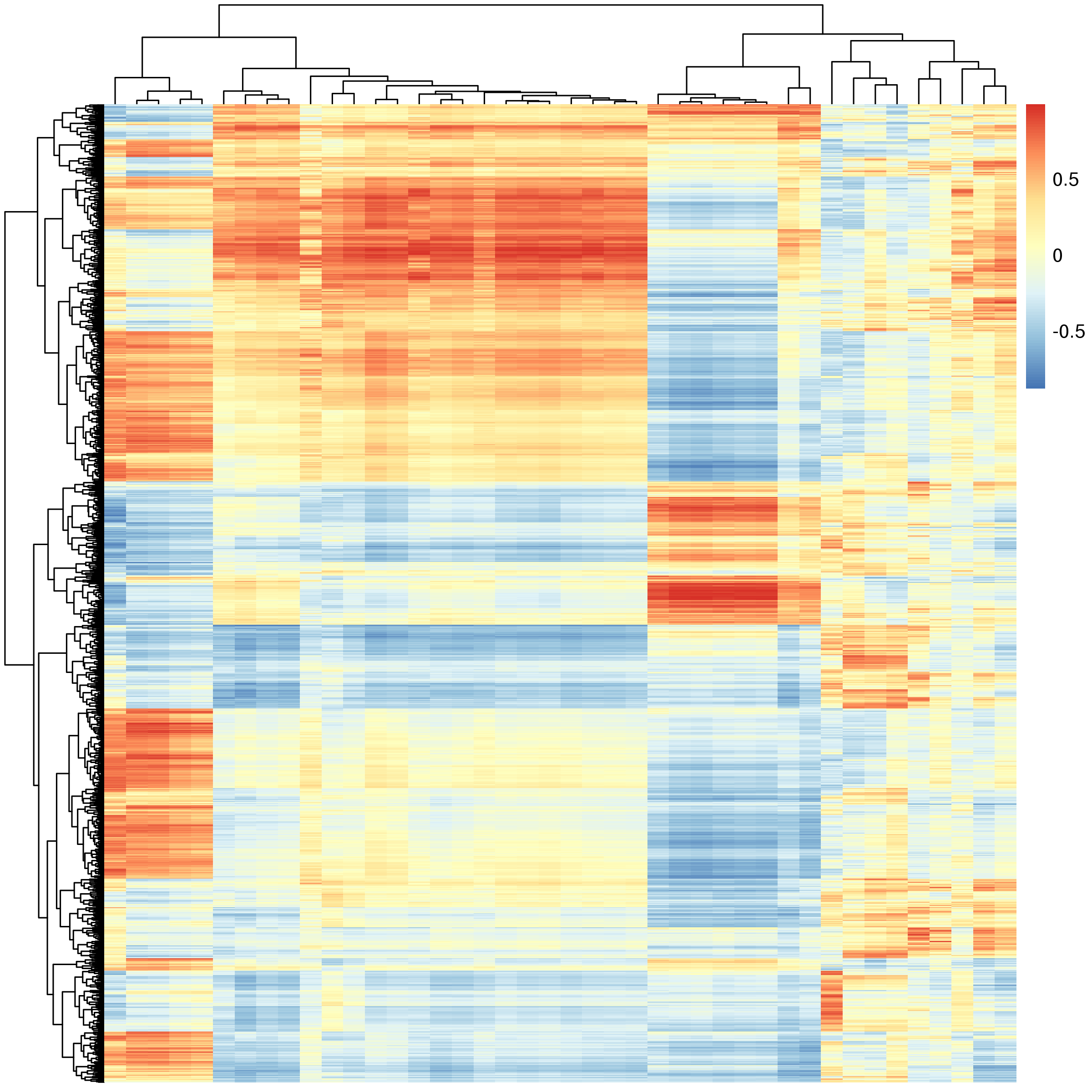


Fig. S2 Correlation analysis between the top 100 DEGs and DAMs. Colors from blue to red indicated the correlation from low to high.
